# Distribution of preoperative angle alpha and angle kappa values in patients undergoing multifocal refractive lens surgery based on a positive contact lens test

**DOI:** 10.1007/s00417-021-05403-w

**Published:** 2021-09-28

**Authors:** Philipp B. Baenninger, Janosch Rinert, Lucas M. Bachmann, Katja C. Iselin, Frantisek Sanak, Oliver Pfaeffli, Claude Kaufmann, Michael A. Thiel

**Affiliations:** 1 Department of Ophthalmology, Cantonal Hospital of Lucerne, CH-6000 Lucerne-16, Switzerland; 2 University of Zurich, Medical Faculty, Zurich, CH-8091 Switzerland; 3 Medignition Healthcare Innovations, Verena Conzett-Strasse 9, PO 9628, CH-8036 Zurich, Switzerland

**Keywords:** Decision-making, Angle alpha, Angle kappa, Multifocal intraocular lens, Refractive lens surgery

## Abstract

**Purpose:**

To assess the preoperative objective angle alpha and angle kappa measurements of patients deciding to undergo multifocal refractive lens surgery based on a subjective positive multifocal contact lens test (MCLT).

**Methods:**

Retrospective, consecutive case series. Alpha and kappa angles were measured using the iTrace aberrometer. All patients also performed a 1-week MCLT. Only patients with a positive MCLT underwent surgery. Visual outcome (UCVA) was obtained in the 1-year follow-up. We assessed the preoperative distribution of angle values within MCLT positive and negative patient groups.

**Results:**

Two hundred seventeen eyes (111 patients) were included. Mean age was 56.4 years (SD 5.6) and 46.9% were female. In 71 eyes (38 patients), MCLT was positive. Of them, 12 eyes (17%) had an angle alpha and angle kappa ≥ 0.5mm. Of 146 eyes (73 patients) who refrained from surgery due to a negative MCLT, 71 eyes (48.6%) had both angles small (<0.5mm). In the 1-year follow-up, UCVA improved by 0.68 logMAR (SD 0.51; *p*<0.001) from baseline. Eyes with both small angle alpha and kappa sizes improved by 0.78 logMAR (SD 0.56), as did eyes with high (≥0.5mm) angle sizes (0.82 logMAR (SD 0.53). UCVA of eyes (*n*=24) with high alpha but low kappa sizes improved less (−0.31 logMAR (SD 0.13; *p*=0.019)).

**Conclusion:**

Four out of five patients with a positive MCLT also had correspondingly small angle values. One-half of patients with low preoperative angle values refrained from surgery due to a negative MCLT result. One-year visual acuity improvement was substantial and independent from angle sizes.

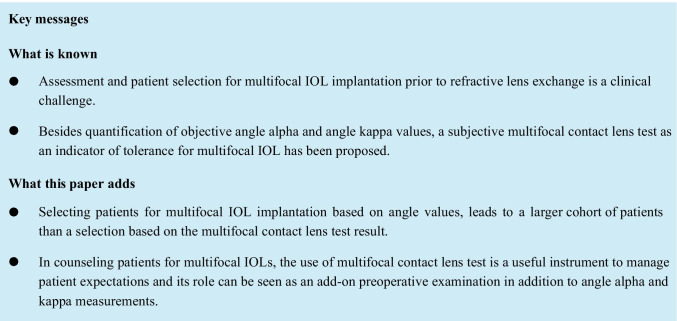



## Introduction

Multifocal intraocular lenses (IOL) are increasingly used in the management of presbyopia treatment [1]. Most patients achieve an improvement not only in distance but also near visual acuity leading to higher spectacle independence and patient’s quality of life [2]. Assessment and patient selection for multifocal IOL implantation is a clinical challenge [3]. Some patients complain about disturbing side effects such as glare and halo phenomena [4]. Research into causes of a poor visual outcome identified a large deviation between the visual axis, pupillary axis, and the optical center of the multifocal IOL as important clinical parameters. Extreme values of these parameters lead to higher order aberrations resulting in decreased visual quality [5]. As a result, ophthalmologists called for an objective preoperative measurement to allow the identification of patients with an increased risk for postoperative glare and halo. Recently, Karhanova and colleagues proposed measuring the angle kappa and angle alpha in preoperative examinations [6]. From the analysis of a smaller sample, Fu and colleagues suggested to select patients for multifocal IOL implantation if they presented with an angle alpha or angle kappa distance smaller than 0.5mm [2].

Preoperative consultations ask for a careful analysis of patient’s lifestyle and expectation as well as thorough examination [1, 7]. As an alternative or add-on examination, preoperative multifocal contact lens test as an indicator for tolerance of multifocal IOL has been proposed [8, 9]. While some anecdotal evidence suggested that a preoperative multifocal contact lens test (MCLT) could be useful to support patients’ decision-making for multifocal IOL surgery, little is known about the relationship between a positive MCLT and the corresponding alpha or kappa angle values. From a theoretical viewpoint, the concordance between the two approaches should be rather high, as patients with large angle alpha and angle kappa would not tolerate the test very well, while patients with normal angle values would. However, the extent to which these theoretical considerations translate into real-world practice is unclear. Therefore, this study assessed the distribution of preoperative objective angle alpha and angle kappa measurements of patients deciding to undergo multifocal refractive lens surgery based on a positive MCLT. We also correlated postoperative visual outcomes to preoperative objective angle alpha and angle kappa sizes. Finally, we investigated whether patients undergoing surgery despite alpha and kappa angles larger than 0.5mm had poorer visual outcomes than patients with lower angle values.

## Methods

### Study design

This retrospective consecutive case series comprised patients being assessed for a refractive lens exchange with implantation of multifocal IOL (AT LISA, Zeiss) at the Cantonal Hospital of Lucerne, Switzerland. All patients underwent a preoperative assessment including a clinical examination as well as subjective refraction, topography, biometry, and aberrometry. After initial consultation with always the same surgeon (PB), all patients underwent a 1-week multifocal contact lens trial. Patients were operated only if the MCLT was positive. The MCLT was defined positive if patients reached a visual acuity ≤ 0.1 (logMAR) and were comfortable to decide to undergo surgery on the basis of their visual experience during the 1-week test period. All patients were examined 12 months after surgery regarding visual acuity and subjective refraction.

The study was conducted according to the standards of good clinical practice and the ethical principles for medical research involving human subjects as outlined in the Declaration of Helsinki. The relevant ethics committee of Lucerne reviewed the protocol of this study and found that this study did not fall under the Swiss Human Research Act. All patients provided written consent to participate in the study.

### Study population

Inclusion criteria were age between 45 and 75 years, willingness to perform the MCLT, and the ability to communicate clearly. Exclusion criteria were irregular corneal astigmatism or manifest regular astigmatism > 2.5 diopter, a history of ocular surface surgery or trauma, intraoperative or postoperative complications, and other ocular pathology that might reduce visual acuity (e.g., corneal disease, glaucoma, amblyopia). To obtain a group undergoing refractive lens exchange, we excluded patients with clinically relevant, age-related cataract, defined as a best-corrected visual acuity > 0 (logMAR) and a dysfunctional lens index ≤ 5.7 [10] in order not to negatively influence the MCLT. We also excluded patients currently wearing multifocal contact lenses, as these patients would not undergo a contact lens test prior to surgery.

### Multifocal contact lens test

The extended-wearable multifocal lens (Alcon AirOptix plus Hydra Glyde Multifocal 8.6/14.0) was fitted according to the manufacturer’s guidelines. These contact lenses use a near center design and addition (Low, Med, High) was chosen on base of patient age. Optimized prescription of contact lenses was achieved by topographic assessment (using Pentacam) as well as subjective manifest refraction. We picked the contact lens based upon the spectacle prescription and used this, if the vision met the standards described. A manifest astigmatism of > 0.75 diopter was corrected with a toric multifocal contact lens (Saphir RX Multifocal Toric, MarkEnnovy). In the toric multifocal contact lens, we used the distance center design for the dominant and the near center design for the non-dominant eye. A settling time of 15min was allowed before determining the final powers. Contact lens power was optimized by over-refraction if distance visual acuity of 0 or less (logMAR) and near visual acuity of 0.1 or less (logMAR) at 40cm was not reached. In such rare cases, a contact lens change was performed. Toric contact lenses were evaluated for rotation. As none of the patient was wearing currently contact lenses, all of them were fitted with extended-wear contact lenses allowing them to wear them day and night for 7 days. Patients were asked to wear contact lenses during an average week with their habitual activities. All patients were instructed to use artificial eye drops four times daily (Refresh Contacts, Allergan) while wearing contact lenses. On day 7, patients came into the clinic, where contact lenses were removed without further assessment of visual acuity and patients asked if they want to go ahead with surgery based on their visual experience with the contact lenses or not. Subjective factors regarding contact lens wear such as comfort or dryness were specifically excluded from the decision-making process.

### Preoperative and postoperative examinations

All patients underwent a comprehensive ophthalmologic examination preoperatively and postoperatively performed by the same experienced ophthalmologic surgeon. Preoperative examinations included measurement of uncorrected (UDVA) and corrected (CDVA) distance and contact lens corrected distance (CLDVA) visual acuities, spherical equivalent, slit-lamp anterior segment evaluation, keratometry, and retina evaluation with dilated pupils. We did not assess the presence of dry eye disease systematically with standardized methods such as OSDI, tear osmolarity, or Schirmer’s test.

The IOL power calculation was performed with the IOL Master biometer (Carl Zeiss Meditec AG) using the Haigis formula with emmetropia as target refraction.

Postoperative examinations included measurements of the UDVA under photopic conditions using Snellen visual charts and then converted into logarithm of the minimum angle of resolution (logMAR) notation. At 3 months, we also assessed patients’ satisfaction asking the patient whether they would recommend this treatment to family and friends.

### Angle kappa and angle alpha measurements

The preoperative and postoperative aberrometry examinations were performed under the same mesopic lighting conditions. The iTrace aberrometer (Tracey Technologies) was used to measure angle alpha and angle kappa as well as dysfunctional lens index. The device calculates wavefront aberration data based on ray-tracing aberrometry and corneal topography, allowing analysis of visual quality. The aberrometer captures an iris image through an infrared camera to display the center of the pupil, the center of the visual axis, and the center of the limbus. Angle kappa measured by the aberrometer is defined by the radial distance between the center of the pupil and the visual axis, estimated by the center of the first Purkinje reflex. Angle alpha is defined by the radial distance between the center of the limbus and the visual axis. The mean of three taken measurements per eye is reported.

### Intraocular lens

The trifocal IOL AT LISA tri 839MP from Carl Zeiss Meditec is a preloaded, ultraviolet-filtering, four-haptic lens, with an overall diameter of 11.0 mm and an optical zone of 6.0 mm.

A corneal astigmatism of > 0.75 diopter was corrected with the toric AT LISA tri toric 939MP. Both intraocular lenses are made of hydrophilic acrylic material and have hydrophobic surface properties. They have a single-piece diffractive multifocal design and the edge of the posterior optical zone is frosted to reduce potential edge glare effects. The near add is 3.33 diopter (D), and the intermediate add is +1.66 D, both calculated at the IOL plane.

### Surgical technique

All surgeries were performed by the same experienced surgeon (PB). A 5.3-mm continuous curvilinear capsulorhexis as well as nuclear softening was made with the Catalys Femtosecond Laser (Johnson&Johnson). After a 2.4-mm superior transparent limbal incision (at 100 degrees) and two 1.0-mm paracentesis 90 degrees apart from each other were created, the O3 (Oertli Instruments) was used for phacoemulsification. The IOL was implanted in the capsular bag. Postoperatively, the patient received topical corticosteroids (Maxidex, Novartis) four times daily with reducing one drop per week for 4 weeks.

### Statistical analysis

We summarized continuous variables with means and standard deviations (SD) and dichotomous variables with percentages. We tested for significance using parametric and non-parametric methods as appropriate. We plotted the spherical equivalent vs. angle kappa values for the patients with positive vs. negative contact lens test and displayed the non-linear relationship using local polynomial smoothing. To compare this relationship with data from published literature, we computed mean values across diopter ranges of spherical equivalent and transformed angle kappa values of our analysis to fit the published data obtained with a different device (Synoptophore). We performed no formal sample size calculation, instead we analyzed all available data to optimize precision of our estimate. Analyses were performed using the Stata 16.1 statistics software package (StataCorp. 2019. Stata Statistical Software: Release 16 College Station, TX: StataCorp LP).

## Results

### Patients’ characteristics

During August 2015 to October 2019, we consecutively enrolled all patients (217 eyes of 111 patients) presenting for refractive lens exchange. Mean patient age was 56.4 years (SD 5.6; range 46 to 72). Overall, 52 out of 111 participating patients (46.9%) were female without difference between the two groups (*p*=0.937). Table 1 shows preoperative patient parameters according to the success of the contact lens test. In terms of subjective preoperative spherical refraction (−0.79 D (*p*=0.104)), no difference between the two groups was found. Dysfunctional lens index showed a clearer lens in the contact lens negative group (median 9.3 (interquartile range (IQR) 8–10) vs. median 8.3 (IQR 6.7–9.5)) in the contact lens positive group; *p*=0.0026. There was no loss of follow-up, and all patients were included in the final data analysis. The 3 months evaluation revealed that all patients would recommend this treatment to family and friends.

**Table 1:** Preoperative patient parameters in both group of patients (contact lens test positive and contact lens negative)

§§	Positive contact lens test (*n*=71)	Negative contact lens test (*n*=146)	
Parameter	Mean ± SD	Mean ± SD	*p* value
UDVA (logMAR)	0.69 (0.50)	0.56 (0.46)	0.046
CDVA (logMAR)	−0.08 (0.08)	−0.11 (0.10)	0.084
CLDVA (logMAR)	0.03 (0.09)	0.03 (0.13)	0.878
Spherical equivalent (D)	1.05 (4.07)	0.24 (3.02)	0.103
Angle alpha (mm)	0.50 (0.18)	0.49 (0.16)	0.662
Angle kappa (mm)	0.36 (0.16)	0.38 (0.16)	0.349
Higher order aberrations (mm)	0.12 (0.09)	0.14 (0.07)	0.309
Dysfunctional lens index (DLI)	7.87 (2.01)	8.60 (1.72)	0.012
IOL power (D)*	22.31 (5.11)	n.a.	n.a.

^*^All patients with a positive contact lens test underwent refractive lens surgery

*UCVA* uncorrected distance visual acuity, *CDVA* corrected distance visual acuity, *CLDVA* contact lens corrected distance visual acuity, *IOL* intraocular lens, *n.a.* not applicable

### Contact lens test and decision for surgery

All patients underwent a MCLT, but only in 38 patients (71 eyes; 33.6%) MCLT was positive. A toric contact lens was fitted in 36 of 71 eyes (50.7%) in the MCLT positive group, and in 72 of 146 eyes (49.3%) in the MCLT negative group (*p*=0.848). Visual acuity with multifocal contact lenses did not differ between groups (*p*=0.878). All contact lens positive patients decided to undergo multifocal refractive lens exchange (surgery group). Contact lens positive patients had a mean spherical refraction of +1.46 (SD 4.05; range −12.0 to +8.75). A multivariate analysis showed that baseline characteristics were not significantly associated with a positive MCLT. Preoperative angle sizes were not associated with a positive MCLT (angle kappa: *p*=0.349, angle alpha: *p*=0.662). Patients with a subjective negative MCLT did not undergo surgery (no surgery group). Of 106 eyes (67 patients) showing both an angle kappa and angle alpha measurement < 0.5mm, only 35 eyes (20 patients; 29.9%) opted for multifocal refractive surgery due to a positive MCLT.

### Angle alpha and angle kappa

There was no baseline characteristic difference in terms of angle kappa (−0.02 mm (*p*=0.349)) and angle alpha (−0.01mm (*p*=0.662)) in both groups (no surgery vs. surgery), and the preoperative distribution is shown in Table 2. Of 146 eyes (73 patients) with negative MCLT, 71 eyes (48.6%) did not undergo surgery despite an angle alpha and angle kappa of < 0.5mm. On the other hand, 12 eyes (16.9%) with a positive MCLT underwent surgery even with an angle alpha and angle kappa ≥ 0.5mm. We found a strong negative correlation between changes of angle kappa values and axial length (−0.035 (95%CI −0.051 to −0.019); *p*<0.001). For angle alpha values the association had the same direction but did not reach statistical significance (−0.023 (95%CI: −0.049 to 0.004); *p*=0.089).

**Table 2:** Distribution of angle alpha and angle kappa measurement in both group of patients (surgery vs no surgery)

			No surgery (*n*=146)			Surgery* (*n*=71)		# Eyes with ideal alpha and kappa angles
Angle alpha								
			<0.5	≥0.5		<0.5	≥0.5	
	<0.5	#(%)	71 (49)	50 (34)		35 (49)	24 (34)	106 (49)
Angle kappa								
	≥0.5	#(%)	3 (2)	22 (15)		-	12 (17)	

^*^All patients with positive contact lens test underwent surgery

^#^ = number

### Visual outcomes

In the 12 months follow-up, UDVA improved by 0.68 logMAR (SD 0.51; *p*<0.001) from baseline. Overall, mean manifest spherical refraction and mean residual astigmatism was 0.38 (SD 0.51), respectively −0.47 (SD 0.37). Eyes with both small angle alpha and kappa sizes improved by 0.78 logMAR (SD 0.56), as did eyes with high (≥ 0.5mm) angle sizes (0.82 logMAR (SD 0.53). Interestingly, 24 eyes with high alpha but low kappa sizes improved significantly less than average (−0.31 logMAR (SD 0.13); *p*=0.019).

### Correlation of spherical equivalent and angle kappa

**Fig. 1:**
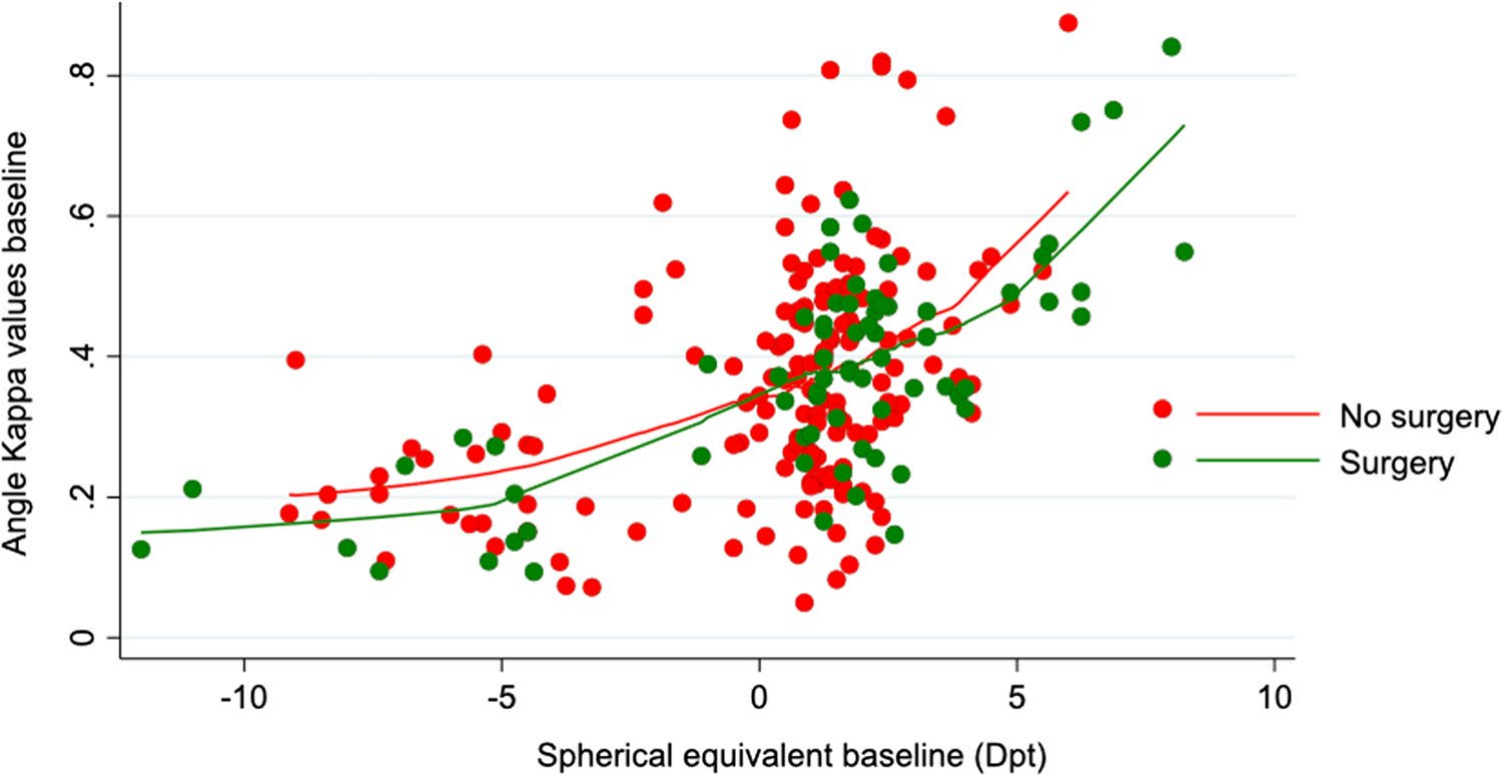
Scatter plot showing the correlation between angle kappa values and spherical equivalent stratified for eyes undergoing IOL surgery (green dots) vs. no surgery eyes (red dots). Lines represent non-linear relationship between plotted variables using local polynomial smoothing

Figure 1 demonstrates the distribution between angle kappa and spherical equivalent in the surgery (positive MCLT) and no surgery (negative MCLT) groups. While the relationships were similar between the two groups, data was more scattered in the no surgery group—the scatter being most pronounced among emmetropic eyes. The relationship between angle kappa values at baseline and spherical equivalent was linear within specific ranges of the spherical equivalent, showing a small slope across smallest values, larger slope between −5 diopters and +5 diopters and highest slope > 5 diopters.

## Discussion

### Main findings

Four out of five patients with a positive MCLT also had correspondingly small alpha and kappa angle sizes. Approximately one-half of patients with low preoperative angle values refrained from surgery due to a negative MCLT result. The 1-year improvements of uncorrected visual acuity in patients who underwent surgery were substantial. In this group of patients undergoing surgery due to a positive MCLT result, improvements of visual acuity did not correlate with angle alpha or kappa sizes.

### Results in context of the existing literature

In our study, patient selection for multifocal lens implantation depended on a positive MCLT rather than taking angle alpha and angle kappa measurements into account. This stands in contrast to other studies suggesting that patients with a large (> 0.5mm) angle kappa are prone to postoperative disturbing photic phenomena as multifocal IOLs may induce more aberrations, glare, and halos [6, 11]. Some proposed that in eyes with larger angle kappa, the light might pass through paracentral IOL rings or its edge inducing a functional IOL decentration [12] and therefore, these patients are not suitable for multifocal IOL implantation [2]. Prakash and colleagues [13] reported a significant association between large angle kappa and patient dissatisfaction; however, they also found many patients with large angle kappa sizes who were also asymptomatic. More recently, and in line with our findings, Garzon and co-workers [14] showed that a large angle kappa did not negatively impact visual outcomes after multifocal IOL implantation. This might be related to the good tolerance towards larger angle kappa because of the technical design of the IOL. In terms of the prediction of a successful postoperative outcome after multifocal contact lenses implantation, Sivardeen and colleagues [15] showed no dependence on angle kappa.

The evidence whether angle alpha sizes correlate with the surgical outcome remains inconclusive [16]. Wang et al. [16] reported a significant change of angle kappa after phacoemulsification when compared to angle alpha and suggested angle alpha as a more reliable and stable factor for preoperative evaluation. Angle alpha can be used to indicate IOL decentration, its impact on visual acuity and optical aberrations is still under debate but likely depends on the design and power of an IOL [17]. For low-power, spherical IOLs, the impact of these decentrations probably is minimal, but for higher power and multifocal IOLs, IOL decentration relative to the visual axis may produce visually significant effects.

There is evidence that angle kappa is a function of the refractive error in normal population with larger values in hyperopic eyes [18–21]. We observed the same correlation with a local linear rise in angle kappa size and hyperopic refraction. As angle kappa can be explained by an anatomic displacement of the fovea from its usual position[22], we hypothesize that angle kappa is a surrogate of the anatomy as well as refraction. This goes in hand with our study finding showing a weak concordance between angle kappa and the MCLT.

### Strengths and limitations

To the best of our knowledge, this is the first study comparing the distribution of preoperative objective angle alpha and angle kappa measurements in patients deciding to undergo multifocal refractive lens surgery based on a positive MCLT.

What are the limitations of this study? First, the retrospective method and small sample size limit the clinical usefulness of this study. Therefore, we call for a validation using prospective data collection and sufficient size to confirm our findings. Second, it can be argued that multifocal contact lenses on the cornea cannot be compared to intraocular lenses due to position on the optical axis, technical design differences, and the possibility of movement in contact lenses. Therefore, there might have been a selection bias with a higher dropout of potential candidates for lens surgery due to a negative experience in the MCLT. In the literature, multifocal contact lens fitting success has been reported to be 44% [23] after 1 week and 57% [24] after 2 months. The fact that all patients in this study were contact lens neophytes might have caused our lower retention rate (33.6%). However, as patient selection is important and proper patient education the key to success and patient’s satisfaction, the MCLT may be useful to simulate unwanted effects and evaluate how well potential candidates can accept them. Although the relationship between a positive MCLT and positive clinical outcome remains poorly understood, it can be hypothesized that patients with positive MCLT have a better neuroadaption and therefore tolerate unwanted effects better. Third, the lack of data on normal angle alpha and angle kappa distribution in the Swiss population impeded us to find out if this study group represents a special selection or the general population. Finally, there was no systematic assessment of patient satisfaction or disturbing photic phenomena in all patients who underwent surgery. Therefore, we were unable to assess differences between patients with low vs. high angle alpha and angle kappa values. However, in a 3-month follow-up assessment conducted routinely at our clinic, all patients who underwent surgery stated that they would undergo surgery again, irrespective of angle alpha and angle kappa values.

### Implications for practice and further research

**Fig. 2:**
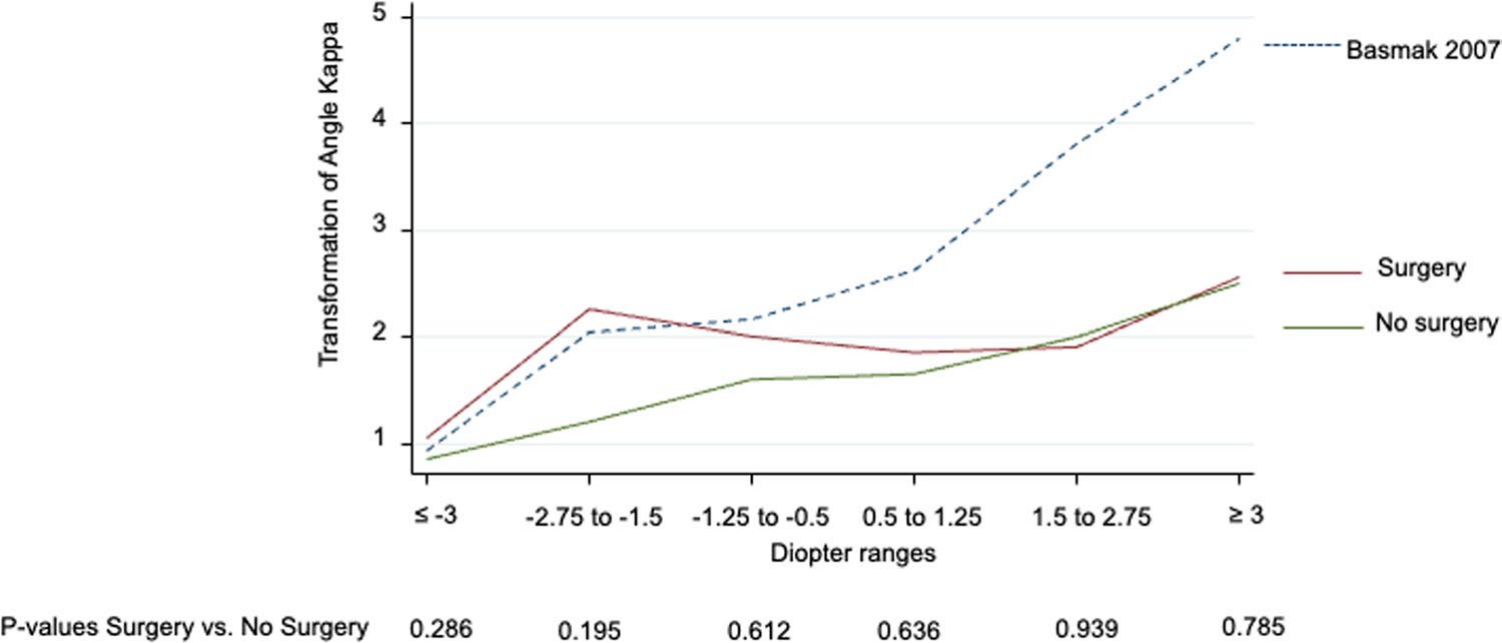
Shows the relationship between spherical equivalent and angle kappa values of this study and the published literature (Basmak 2017, Reference 18 of this article). For the purpose of comparability, mean spherical equivalent values were calculated for intervals used by Basmak et al. and the kappa values of this study were transformed

In this study, postoperative satisfaction with visual outcome was not correlated with the size of angle alpha and angle kappa. We argue that angle alpha and angle kappa are anatomical surrogates of refraction as there was a clear correlation between these parameters both in our study and a published report [18] (see Fig. 2). Key to success in patient selection will always be the understanding of patient’s lifestyle needs and visual expectations. Whether or not the MCLT identifies a patient group who will be more satisfied with a postoperative result than a group selected based on angle alpha and or kappa values needs to be examined. The finding of this study that eyes with high alpha but low kappa sizes improved significantly less than average remains ill understood and needs to be confirmed in further research.

### Conclusions

Patients with a positive MCLT typically show small angle alpha and kappa sizes. In this study, many patients with small angle sizes refrained from surgery due to a negative MCLT. Selecting patients for multifocal IOL implantation based on angle values leads to a larger cohort of patients than a selection based on the MCLT result. In counseling patients for multifocal IOLs, we believe that the use of MCLT prior to refractive lens exchange is a useful instrument to manage patient expectations. Its role can be seen as an alternative or add-on preoperative examination in addition to angle alpha and kappa measurements. However, it is time consuming and does not represent a 1:1 experience of the implanted multifocal IOL. Furthermore, the use of contact lenses is limited to a clear crystalline lens, but multifocal IOLs are mostly implanted in patients with cataracts.

## Data Availability

The datasets used and/or analyzed during the current study are available from the corresponding author on reasonable request.
